# Validation of the WHO self-reporting questionnaire-20 (SRQ-20) item in primary health care settings in Eritrea

**DOI:** 10.1186/s13033-018-0242-y

**Published:** 2018-10-24

**Authors:** Tesfit Brhane Netsereab, Meron Mehari Kifle, Robel Berhane Tesfagiorgis, Sara Ghebremichael Habteab, Yosan Kahsay Weldeabzgi, Okbazghi Zaid Tesfamariam

**Affiliations:** 1 School of Public Health, Asmara College of Health Sciences, Asmara, Eritrea; 2 Ministry of Health, Asmara, Eritrea

**Keywords:** Common mental disorder, Self reporting questionnaire (SRQ-20), Primary health care, Eritrea

## Abstract

**Background:**

In Eritrea, highly centralized mental health care services and lack of trained psychiatric personnel at primary health care units remain a challenge to the mental health care system. These problems can be minimized by introducing screening programs with a simple screening tool for mental disorders in the primary health care settings. Thus, this study aimed to assess the validity of the WHO self-reporting questionnaire 20 (SRQ-20) in Tigrigna version for use in Eritrean primary health care setting.

**Methods:**

The SRQ-20 was translated into a local language (Tigrinya) in a process of forward and backward translation. SRQ-20 data were collected in a primary health care setting on 266 respondents. Internal reliability was tested using Cronbach’s alpha. Factorial validity was done using principal component analysis with varimax rotation to investigate whether SRQ-20 items properly measure the underlying dimensions of mental illness. Criterion validity was analyzed by looking at the relationship between the SRQ-20 and Brief Psychiatric Rating Scale using Pearson’s correlation coefficient. Sensitivity, specificity and the predictive values of the screening instrument were used to assess how well the results of SRQ-20 correspond with the criterion instrument.

**Results:**

The SRQ-20 had good internal reliability (α = 0.78). Factor analysis yielded two factors, explaining 31.2% of the total variance. The instrument performed well in detecting common mental disorders, with an area under the curve (AUC) of 0.879 (SE = 0.23, 95% CI 0.83–0.92) to the overall sample and with optimal cut-off score at 5/6 with sensitivity 78.6% and specificity 81.5%. Cut-off scores were different for women (5/6) and men (4/5). For male participants, the AUC statistic was 0.877 (SE = 0.04, 95% CI 0.79–0.96) and 0.871 (SE = 0.02 95% CI 0.81–0.92) for female participants.

**Conclusion:**

The Tigrinya version of the SRQ-20 can be used for screening probable common mental disorders in Eritrean primary health care setting, but cut-off scores need to be adjusted for men and women separately.

## Background

Common mental disorders refer to two main diagnostic categories: depressive disorders and anxiety disorders. These disorders impact on the mood or feelings of affected persons; symptoms range in terms of their severity (from mild to severe) and duration (from months to years). These disorders are diagnosable health conditions, and are distinct from feelings of sadness, stress or fear that anyone can experience from time to time in their lives. Anxiety disorders involve generalized mood condition involving feelings of uneasiness, being fearful or feeling nervous whereas depressive disorders have emotions that involve feeling low, sad, fed up or miserable [1, 2].

Untreated mental disorders result in poor outcomes for co-morbid physical illness commonly seen in the primary health care settings. Moreover, patients with mental disorders have a heightened risk of suffering from physical illness because of diminished immune function, poor health behavior, non-compliance with prescribed medical regimens and barriers to obtaining treatment for physical disorders.

The fundamental mental health facts report has underscored the seriousness and increasing prevalence of mental health problems, highlighting that, every week, one in six adults experience symptoms of a mental health problem [3]. Moreover, a recent index of 301 diseases found mental health problems to account for 21.2% of years lived with disability worldwide, indicating mental health diseases are becoming important contributors to the global burden of disease [4]. The consequences of mental disorders in terms of lost health are significant. Depression is ranked by WHO as the single largest contributor to global disability (7.5% of all years lived with disability in 2015) while anxiety disorders are ranked 6th (3.4%) [5].

Mental illness is considered a silent epidemic throughout most parts of Africa. Because of structural and systemic barriers such as inadequate health care infrastructure, insufficient number of mental health specialists, and lack of access to all levels of care, mental illness has been characterized as a neglected and increasingly burdensome problem affecting all segments of the population throughout Africa. Prioritizing mental health has also been difficult due to lack of resources, limited funding coupled with absent or ineffective mental health policies [6].

A recent national mental health survey conducted in Eritrea reports a common mental disorder prevalence rate of 14.5% in the general population [7]. The 2015 global burden of disease study reports 219, 549 cases of depressive disorders (4.3% of the total population) and 156, 599 cases of anxiety disorders (3.1%) in the country [8]. The consequences of these disorders in terms of lost health are also considerable. The 2015 global health estimate for Eritrea attributes 40, 426 total years lived with disability for depressive disorders (8.2% of years lived with disability) and 14, 474 total years lived with disability for anxiety disorders (2.9%) [9].

In Eritrea, inequitable distribution of psychotropic drugs, highly centralized mental health care services to urban centers and lack of trained psychiatric personnel at primary health care units are some of the challenges the mental health program is facing [10]. According to the study of assessment of mental health system in Eritrea, similar drawbacks as in the majority of developing countries are prevalent. These include absence of revision of the mental health policy, very low financial expenditure on mental health and lack of access to mental health facilities for in the country there is only one mental health hospital with its only outpatient unit and the only community residential facility; all concentrated in the capital city, Asmara [11]. It is estimated that the treatment gap (defined as the number of people not receiving treatment despite their need), for Eritrea is nearly 97%. Moreover, there is an acute shortage of skilled mental health professionals and mental health services in large parts of the country [12]. There is also inadequate budget for primary mental health services as most of the budget is used by the only tertiary level psychiatric hospital (St. Mary Psychiatric Hospital).The recent national mental health survey posits that the St. Mary Psychiatric Hospital does not have the capacity to care for all the mentally ill in Eritrea and recommends establishment of mental health care wings, prioritizing the zonal referral hospitals for initial action. The study also underscored the care of the mental ill should be integrated as part of comprehensive primary health care service and be considered with the routine health service delivery activities [7].

The problems of low financial expenditure and inequity in distribution of mental health facility can be minimized by introducing psychosocial interventions especially screening program for mental disorder in the primary health care settings which are accessible and affordable to the general population. Considering the understaffed and undertrained personnel in primary health care settings of the country, the SRQ would play an important role in improving community’s mental health. The WHO SRQ-20 is not functional and has never been used in Eritrea. The culturally sensitive nature of the questionnaire and the language difference of settings necessitate adaption of the instrument locally. The SRQ is expected to help because it is a simple instrument, poverty friendly, timely and does not need a professional to administer; which is feasible in our country.

There are many psychiatric screening instruments developed by the World health Organization (WHO). Nevertheless, these instruments are time-consuming and require trained personnel for administration either through interview or through self-administered questionnaire, being infeasible for use in the primary health care, which, often, are the target settings for screening and early detection of CMDs. This gives rise to the need of the self-reporting questionnaire (SRQ-20), a 20-item mental disorder screening instrument developed by WHO to be applied in patients attending primary health care settings [13]. Trudy Harpham et al. reviewed the performance and use of this questionnaire and concluded that the SRQ-20 as a cost-effective instrument to measure mental health at community level with high face and criterion validity, ease of use and suitability to administer it by a lay health worker [14]. Several comparative and validation Studies also found the SRQ-20 to be preferred from many other screening questionnaires [15–18].

The SRQ-20 is an instrument with twenty simple to understand items which question respondents about symptoms and problems likely to be present in those with neurotic disorder. It includes binary (yes/no) questions only, with codes “1” which represents the presence of a symptom, and “0” if the symptom is absent. The SRQ-20 item questions reflect depression, anxiety and psychosomatic complaints, which are all together, grouped under the common mental disorder (CMD) and have been found to detect probable cases of it with satisfactory accuracy [19]. However, the validity, reliability and cut-off of the SRQ-20 vary in different settings across variety of populations (with different culture, language, setting and gender) [15–19]. For this reason, adapting the SRQ-20 in one’s community setting or population primarily requires validating the screening instrument in one’s own setting. Many developing countries have validated and have been using the SRQ-20 in many community-based screening programs. In Eritrea, the SRQ is not functional to use as screening instrument in PHC, for its validity is not yet assessed.

Therefore, this study aimed to assess the validity of the WHO self-reporting questionnaire 20 (SRQ-20) item in Tigrigna version in Eritrean setting. Specifically, this study evaluated the psychometric properties, optimal gender specific cut-off points for detecting CMDs using SRQ as a screening tool and assessed the SRQ-20 items’ capacity to screen for mental disorder relative to the gold standard (BPRS).

## Methods

### Study design

This study was carried out using a cross-sectional study design.

### Study settings and population

This study was conducted from September to December 2017 in two urban health facilities selected using purposive sampling. The selected health facilities were Semienawi Asmara Health Centre (SAHC) and Godaif Community Hospital (GCH). The health facilities were selected purposively because they serve large number of population and are relatively representative of PHCs in Asmara in terms their location and socio economic diversity. Both health facilities provide primary health care services to their local population and refer to higher health services for specialized care and services.

### Sample size and sampling method

Sample size determination was done on the basis of an estimated common mental disorder prevalence of 50%, as there was no local study done before. At a 95% confidence interval, a total sample of 266 participants were selected using the Daniel and Macfarlane single proportion formula for finite population, n = Z^2^ × P × Q/E^2^. The study respondents were recruited using a simple random sampling. Eligible participants included both male and female who were in the age range of 18–65 years and who were attending the selected health facilities at the time of data collection. Individuals were excluded from the study if they were medically unstable, if they required referral to a higher level of care, or were not willing to participate in the study.

### Data collection instruments

In addition to the demographic characteristics of the respondents such as age, gender, ethnicity, educational level and marital status, the measures utilized in our study include the WHO self-reporting questionnaire and Brief Psychiatric Rating Scale.

### The self-reporting questionnaire

The self-reporting questionnaire is based on the original 20 yes/no questions translated to Tigrinya which consists of 20 items indicative of non-psychotic mental disorders. The questions are related to certain pains and problems that may have bothered the participant in the last 30 days. If the participant thinks the question applies to him/her, then will answer as yes and the other ways it will be no. The translation of these questions included language experts. It was translated in the standard back and forth translation format.

### The Brief Psychiatric Rating Scale

The Brief Psychiatric Rating Scale is a 24-item questionnaire that provides a clinical assessment of the interviewee’s psychiatric status and leads to a psychiatric diagnosis. Although yet to be validated, the BPRS item is frequently used in primary health care settings of Eritrea. However, the instrument has been used in a number of countries with similar context to Eritrea and its factor structure remains to be similar across studies [20]. BPRS requires well-trained professionals to administer it; consequently it is not feasible to use it as a large scale screening instrument limiting its utility [21]. In our study, we used the instrument as the gold standard for detecting common mental disorders in primary health care settings in Eritrea.

### Instrument translation

The WHO SRQ-20 was not functional and has never been used in Eritrea in Tigrigna version. The culturally sensitive nature of the questionnaire and the language difference of settings necessitate adaption of the instrument locally. Prior application of the SRQ-20, cross-cultural adaption (which includes translation into local language) and determination of optimal cut-off scores should be performed [14]. For this study, first all the twenty items of the WHO SRQ-20 were translated to Tigrigna, a widely spoken local language in Eritrea by a psychiatrist and language experts. For the purpose of conceptual meaning adjustment, the instrument was blindly back translated by other language experts and psychiatrists who were not familiar with the purpose of the study. Any inconsistency in language meaning and translation differences were resolved through a focus-group discussion.

### Study procedure

Prior to the interview, each participant was informed about the confidentiality of the data collection process. They were assured that participation was voluntary, and could withdraw from the interview at any time. After fully describing the study objectives in a private room, consented respondents were separately interviewed from other patients for health related questions. The interview was conducted during day time to the patients in the waiting room for outpatient treatment. During the interview, the data collector explained that they will be asked health related questions but not exactly about mental health in order to reduce misleading answers. If the participant was required by the medical personnel in the OPD then the interviewer would temporarily suspend the interview and continue after the patient completed OPD visit.

### Data analysis

All data were analysed using SPSS (Statistical Package for Social Sciences) version 20.

### Reliability

Reliability (internal consistency) of the SRQ-20 questionnaire was calculated using a reliability coefficient (Cronbach’s alpha).

### Face validity

Face validity of the Tigrigna version of SRQ-20 in the pilot study through asking the respondents to guess the aim of the questionnaire at the end of each interview.

### Content and semantic validity

In this study, content validity was done through mental health professionals, psychiatric nursing experts and language experts to judge the content and semantic validity of the instrument after its translation to the local language, Tigrigna.

### Construct validity

To assess factorial validity, we used principal component analysis technique with varimax rotation to investigate whether SRQ-20 items measure properly the underlying dimensions of mental illness. The Kaiser–Guttman criterion was employed to extract factors with Eigen values above one [22, 23]. The scree plots were examined to identify the optimal number of factors for extraction. The Kaiser–Meyers–Olkin (KMO) test was calculated to determine the sampling adequacy for the procedure. Bartlett’s test of sphericity was calculated to confirm if it was significant to perform a PCA.

### Criterion validity

The relationship between the testing instrument, the SRQ and the criterion (the BPRS) was assessed using Pearson’s correlation coefficient. Moreover, sensitivity, specificity and the predicative values of the screening instrument were used to assess how well the results of SRQ-20 correspond with the criterion instrument (BPRS).

The optimum cut-off scores were chosen with maximum sensitivity and reasonable specificity at various cut off points. The Receiver Operating Characteristics (ROC) analysis was performed to evaluate the screening properties of the SRQ-20, using BPRS as the gold standard. The Area under Curve (AUC) statistic was calculated as a measure of accuracy of the SRQ-20.

## Results

In this study, out of the 266 participants, more than half were women (54.5%, n = 145), and about 70% were in the age group of 18–35 years. The mean age and standard deviation of the participants was 32 (± 11.09). Most of the study participants reached high school (35.3%, n = 94) and (62%, n = 167) were married. Tigrigna ethnic group was dominant over other ethnic groups (89%); this is because the setting was in Asmara city in which majority of its population is Tigrigna. Detailed information about demographic characteristics of the study participants can be referred in Table 1.

**Table 1:** Socio-demographic characteristics of study participants by gender

Variables	Total sample n = 266 (%)	Males n = 121 (%)	Female n = 145 (%)
Age			
18–25	94 (35.3%)	28 (29.78%)	66 (70.2%)
26–35	93 (35%)	36 (38.7%)	57(61.29%)
36–45	43 (16.2%)	28 (65%)	15 (35%)
46–65	36 (13.5%)	28 (77.7%)	8 (22.2%)
Mean (SD)	32 ± 11.09		
Ethnicity			
Tigrigna	237 (89.1%)	114 (47.6%)	123 (52.3%)
Tigre	25 (9.4%)	6 (24%)	19 (76%)
Saho	3 (1.1%)	1 (33.3%)	2 (66.6%)
Bilen	1 (0.4%)	0	1
Educational level			
Illiterate	2 (0.8%)	0	2
Elementary	30 (11.3%)	16 (53.3%)	14 (46.6%)
Middle school	79 (29.7%)	20 (25.3%)	59 (74.6%)
High school	94 (35.3%)	39 (41.4%)	55 (58.5%)
12+	61 (22.9%)	38 (62.2%)	23 (37.7%)
Marital status			
Married	167 (62.8%)	66 (39.5%)	101(60.4%)
Unmarried	89 (33.5%)	52 (58.4%)	37 (41.5%)
Widowed	6 (2.3%)	2(33.3%)	4(66.6%)
Divorced	4 (1.5%)	1 (25%)	3 (75%)

### Prevalence of common mental disorders (CMDs)

The prevalence of common mental disorders (CMDs) was calculated using the gold standard questionnaire (BPRS). Out of the 266 total samples, 60 (23%) respondents were diagnosed of CMD. For male and female respondents, 17% and 27% were diagnosed with CMD respectively.

### Psychometric properties of the Tigrigna-version SRQ-20

#### Reliability

Cronbach’s alpha of 0.784 indicated good internal consistency.

#### Face validity

The Tigrigna version SRQ-20 demonstrated good face validity when examined in the pilot study. The participants responded well throughout the study as there were no non-respondent from the approached individuals. This 100% item-completion rate also indicated strong face validity of the Tigrigna-version SRQ-20. Moreover, majority of the participants in the pilot study correctly guessed the aim of the questionnaire and understood that the questions were designed to assess mental status.

#### Content and semantic validity

When translated to Tigrigna all 20 items of the SRQ questionnaire were examined by mental health professionals and psychiatric nursing experts to judge the content validity whether it could be able to detect mental health disorder. The translated version was piloted in twenty respondents to check for conceptual equivalence, acceptability and feasibility. Because of being offensive and sensitive issue, the 17th item was placed to be at the end of the questionnaire. The translation did not change the ability of the questionnaire to detect the symptoms of CMDs. Moreover, some questions were modified to meet the cultural context, in which direct translation of word for word yielded meaningless and deviated understanding of the item. For example, item 7 says, “Is your digestion poor?” which the word “poor” indicates weak. Another question item19, “uncomfortable feeling in the stomach” cause misunderstanding for expecting other feelings rather than common bowel disorders like indigestion, constipation or epigastric pain (Tables 2, 3).

**Table 2:** The 2 × 2 table of SRQ-20 item

Diagnosis by BPRS	Diagnosis by BPRS		Total
	Positive	Negative	
Screened by SRQ			
Positive	47 (true positive/TP)	38 (talse positive/FP)	85
Negative	13 (false negative/FN)	168 (true negative/TN)	181
Total	60	206	266

**Table 3:** Sensitivity and specificity of SRQ at various cut-off points

Cut-off score	Total sample		Male		Female	
	Sensitivity (%)	Specificity (%)	Sensitivity (%)	Specificity (%)	Sensitivity (%)	Specificity (%)
2/3	98.3	39.8	95.2	49	100	31
3/4	95	56.8	85.7	67	100	48
4/5	90	70	85.7	74	92.3	65
5/6	78.6	81.5	76.2	84	82.1	73.6
6/7	73	85	71.4	91	76.9	78.3
7/8	58.3	90.8	61.9	95	56.4	86.8
8/9	43.3	94	38	95	46.2	92.5

#### Construct validity

A principal components analysis (PCA) was conducted on the SRQ-20 data with varimax rotation. The tests of suitability for PCA indicated that this analysis was appropriate. The Kaiser–Meyers–Olkin (KMO) test result of 0.774 confirmed the sampling adequacy for the procedure. Bartlett’s test of sphericity was significant indicating that it was appropriate to perform a PCA.

Using a combination of the Kaiser–Guttman criterion and the scree plot method we decided to extract two factors for the entire sample which explained 31.2% of the variance in the sample. We labeled these constructs factors into 1 and 2. Factor 1 included difficulty thinking clearly, tired easily, always tired, worthlessness, unhappy feeling, excessive crying, shaking hands, uncomfortable stomach, work suffering, difficulty in decision, stress simply, headache, fear easily, sleep badly, indigestion after eating, poor appetite and suicidal thought. Factor 2 included: difficulty in thinking, tired easily, always tired, worthlessness, stress simply, headache, loss of interest, difficulty in social interaction and suicidal thought. Other items were excluded from both factors as they had low factor loadings (see Table 4).

**Table 4:** Factor loadings of the principal component analysis in the total sample

Items	Factor 1	Factor 2
Thinking properly	*0.665*	*0.365*
Easily tired	*0.575*	*−* *0.403*
Always tired	*0.565*	*−* *0.547*
Worthlessness	*0.552*	*0.457*
Good feeling	*0.528*	0.165
Excessively crying or attempt to it	*0.522*	0.017
Tremor	*0.521*	0.211
Uncomfortable stomach	*0.507*	− 0.222
Suffering	*0.483*	0.246
Decision	*0.478*	0.181
Stress simply	*0.458*	*−* *0.354*
Headache	*0.454*	*−* *0.396*
After eating	*0.371*	− 0.111
Loss interest	0.265	*0.390*
Difficulty social work	0.263	*0.357*
Enjoy	0.244	0.256
Fear simply	*0.456*	− 0.108
Appetite	*0.374*	− 0.196
Sleep	*0.432*	− 0.291
Suicidal thought	*0.343*	*0.343*

Factor loadings > 0.3 are in italics text

#### Criterion validity

**Fig. 1:**
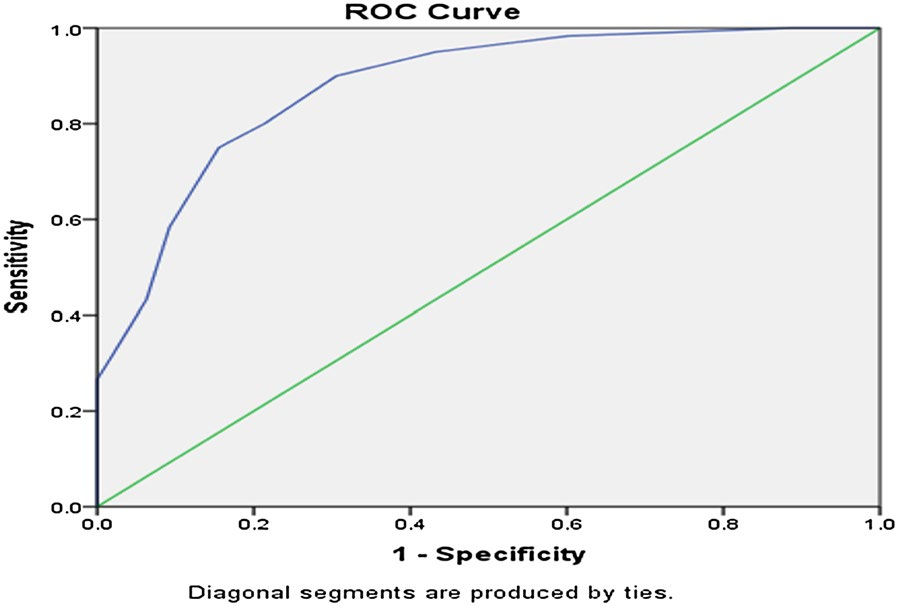
Receiver operating curve for the total sample. AUC = 0.879 (SE = 0.23, 95% CI 0.83–0.92)

**Fig. 2:**
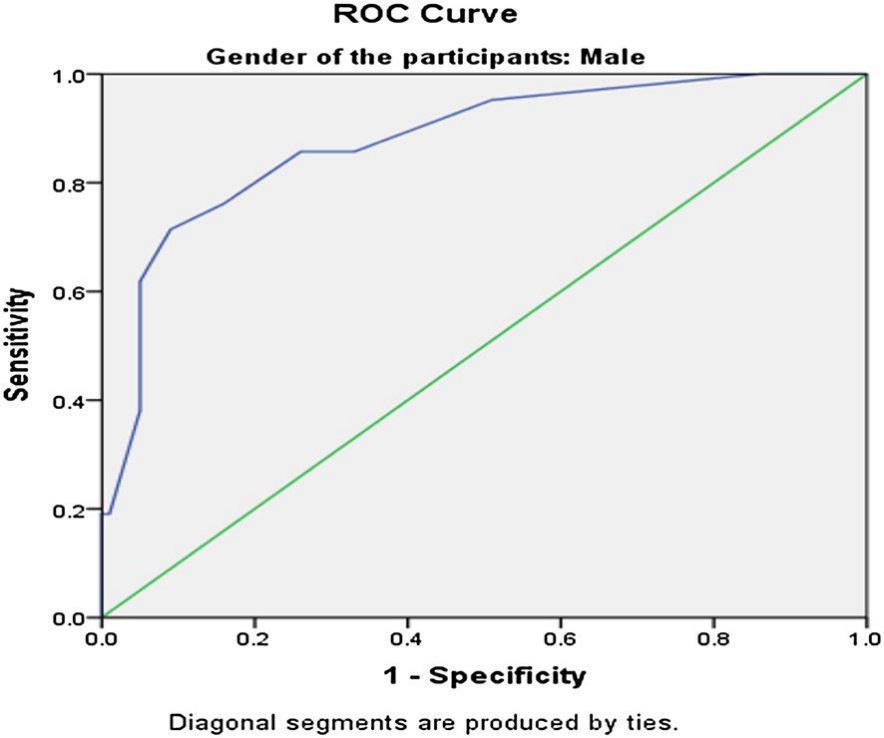
Receiver operating curve for male participants. AUC = 0.877 (SE = 0.04, 95% CI 0.79–0.96)

**Fig. 3:**
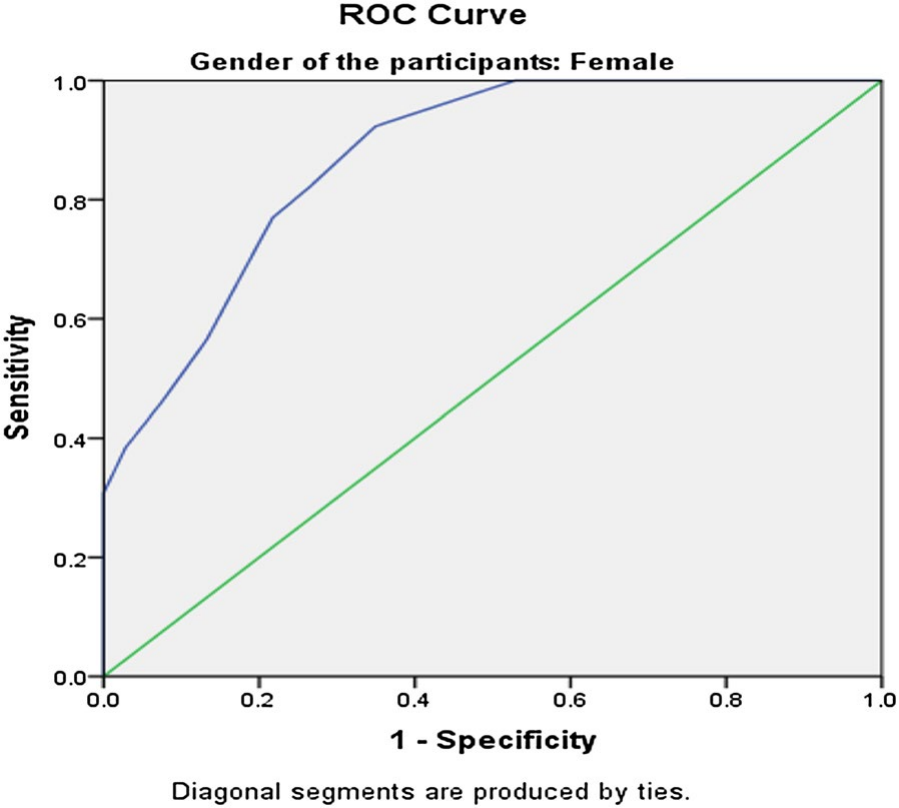
Receiver operating curve for female participants. AUC = 0.871 (SE = 0.02 95% CI 0.81–0.92)

Both the gold standard questionnaire (BPRS) and the screening instrument Tigrigna version of the SRQ-20 were found to be moderately correlated(r = 0.537) but statistically highly significant with a p-value of < 0.001. As shown in Fig. 1, the receiver operator characteristic (ROC) curve analysis for the total sample revealed an area under the curve (AUC) statistic of 0.879 (SE = 0.23, 95% CI 0.83–0.92).This demonstrates the SRQ-20 is highly effective in detecting mental disorders. When analyzed separately for men and women, the SRQ-20 showed to perform well. For male participants (Fig. 2), the AUC statistic was 0.877 (SE = 0.04, 95% CI 0.79–0.96) and the AUC statistic for the female participants (Fig. 3), was 0.871 (SE = 0.02 95% CI 0.81–0.92).

**Fig. 4:**
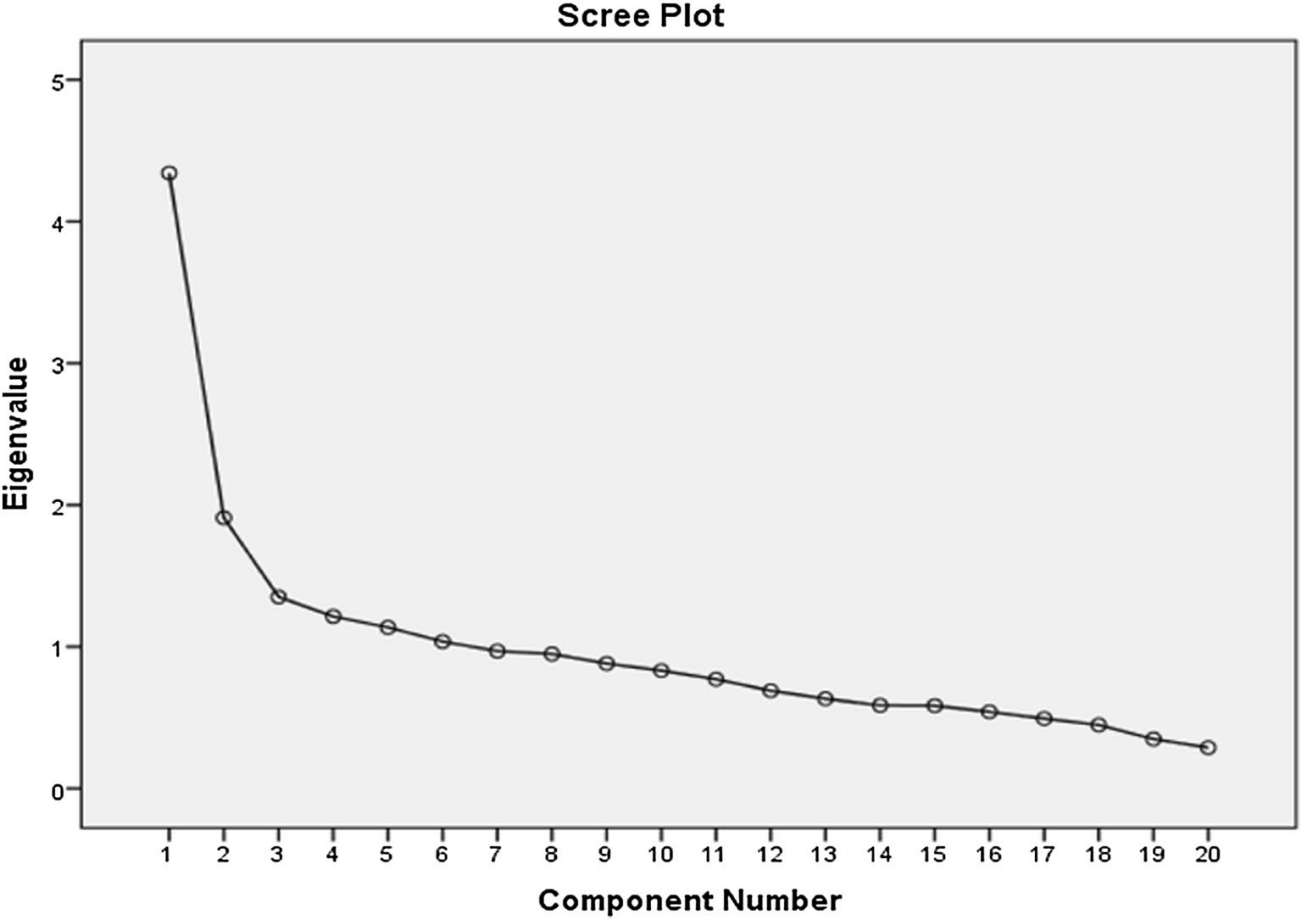
Scree plot of the SRQ-20 components

If the SRQ-20 is to be effective in detection of CMDs, it should have a balanced high sensitivity and specificity with optimum cut-off point scores obtained through the ROC analysis. This balance was achieved at 5/6 optimal local cut-off point with sensitivity 78.6% and specificity 81.5% for the overall sample in our study. There exhibits a difference in the optimal cut-off between male (4/5) and female (5/6) respondents (Fig. 4).

The positive (PPV) and negative predictive values (NPV) of the Tigrigna version SRQ-20 were also calculated. The NPV, 92.8% is considered good indicating high ability of CMD non-cases detection and PPV was found to be 55.3%.

These are the measures of determining the validity of the scale for screening of the PPD.

$$\begin{aligned} {\text{Sensitivity}} & = {\text{TP}}/{\text{TP}} + {\text{FN}} = 47/\left( {47 + 13} \right) \hfill \\ & = 0.7833 = 78.3\% \hfill \\ \end{aligned}$$

$$\begin{aligned} {\text{Specificity}} & = {\text{TN}}/{\text{TN}} + {\text{FP}} = 168/168 + 38 \hfill \\ & = 0.8155 = 81.5\% \hfill \\ \end{aligned}$$

$$\begin{aligned} {\text{Positive}}\;{\text{predictive}}\;{\text{value}}\;\left( {\text{PPV}} \right) & = {\text{TP}}/{\text{TP}} + {\text{FP}} \hfill \\ & = 47/\left( {47 + 38} \right) = 0.552 = 55.2\% \hfill \\ \end{aligned}$$

$$\begin{aligned} {\text{Negative}}\;{\text{predictive}}\;{\text{value}}\;\left( {\text{NPV}} \right) & = {\text{TN}}/{\text{TN}} + {\text{FN}} \hfill \\ & = 168/\left( {168 + 13} \right) = 0.987 = 92.8\% \hfill \\ \end{aligned}$$

## Discussion

This study aimed to validate the SRQ-20 item in Eritrean primary health care settings. In the overall sample, male to female ratio was almost proportional. Most of the respondents were in the age range of 18–25 years, married, Tigrigna ethnicity and middle-schooled. As can be inferred from the findings, the SRQ-20 item was found to have a substantial capacity for screening of common mental disorders in Eritrean primary health care settings. The cut-off scores for the SRQ-20 differed by gender.

In the present study, the prevalence of CMDs in the overall sample was found to be 23%. Many epidemiological studies conducted in primary health settings identify mental disorder using screening instruments or clinical diagnosis [24]. Since the SRQ20 cannot be a substitute for, or equivalent to, a clinical diagnosis [14], we used the BPRS results to measure the prevalence rate of CMDs in our country. As per cross-sectional studies conducted by WHO at 14 sites found out, about 24% of all patients in PHC settings had a mental disorder [24]. The prevalence of CMDs in this study as measured by the gold standard, was higher (23%) than the previous prevalence of mental health disorders in the country, 14.5% in 2014 [25]. The higher prevalence rate attained from the current study with respect to former national prevalence rate, may be associated with a steady increment in the prevalence rate of mental disorders globally especially for the low-income countries as justified by sustained population growth and poverty as strong risk factors [26]. As this study was conducted in PHC settings, where respondents came chiefly to address their underlying health problems, over-reported symptoms of CMDs may had pathologic origin related to it and hence might have contributed to high rate of prevalence. The result from this study appeared to be lower when compared to prevalence of two towns in Ethiopia, 33.6% in Jimma and 32.4% in Kombolcha [27, 28]. According to the WHO reports from 14 countries worldwide, the prevalence of CMD in PHC settings is 24% [24], which is almost similar to the finding in this study. In 2013, a study done in Malawi also reported that CMD prevalence in PHC was 20.1% [29].

Majority of the CMD cases in our study were females when compared to their male counterparts. This is similar to findings from other studies done in Ethiopia and Malawi [27–29]. The reason for this could be as a result of gender disadvantages like social burden and maternity issues. There is also tendency of over-reporting mental problems by females.

In our study, we found good internal consistency of α = 0.78, almost similar with findings in China (α = 0.79) [30], but slightly lower than that of South Africa (α = 0.84) [17].

Similar with other studies, the findings from the present study indicates that the SRQ-20 item is a useful tool in Eritrean primary health care settings. In this study, the area under the ROC curve statistic was 0.87. This result is consistent with findings from other countries’ primary health care settings where the area under the ROC curve ranged from 0.76 in Rwanda to 0.92 in Uganda [31, 32]. In this study, the AUC for women and men were 0.871 and 0.879 respectively. This indicates that the SRQ-20 was just as effective in distinguishing between cases and non-cases in both groups. This screening tool has never been validated in our country’s primary health care settings. But now since the results confirm the performance of the instrument in detecting CMDs, the SRQ-20 can be a suitable tool for measuring CMD in PHC settings of Eritrea.

In this study, the balanced sensitivity and specificity was achieved at the higher cut-off score of 5/6 for women, as compared to the 4/5 cut-off score for men. For overall sample, an optimal local cut-off, 5/6 was found to balance the trade-off between sensitivity and specificity at 78.6% and 81.5% respectively. This is almost similar with a study conducted in the UAE were a cut-off score of 5/6 produced a balanced combination of sensitivity and specificity of 78.3% and 75.2%, respectively [33]. Studies conducted in other countries like Rwanda and South Africa also reported gender differences in SRQ scores with higher cut-off scores for women than men [29, 34]. However, there are few studies conducted in other countries like Zambia and the United Kingdom that have found no differences in the optimal cut-off scores for both sexes [35, 36].

Although the study has come up with important findings, due to some limitations, the findings should be interpreted with caution. As the translated version of SRQ-20 is in Tigrigna, other language speakers from different ethnic groups may not be beneficiaries of the instrument and limited diversity in the ethnicity of the participants may not represent and generalize the result of the current study to all Eritrean communities.

The findings of the study have good implication for addressing the issue of screening for common mental disorders in primary health care settings. Here, the SRQ-20 item has been found to be suitable in primary a health care setting which is simple, easy to administer and with a yes/no questionnaire which is convenient for the interviewer and the respondents. Due to resource-poor conditions with ineffective means of detecting CMDs in Eritrea, the Tigrigna-version SRQ-20 item is expected to contribute in reducing the prevalence and burden of CMDs through its simplicity, acceptability and feasibility.

## Conclusion

The Tigrigna-version SRQ-20 is a valid and reliable instrument which has the capacity to detect cases of CMDs in cost-effective manner. The use of the SRQ-20 is recommended for use in the PHC settings for screening probable common mental disorders in Eritrea.
